# Diverse Regulatory Cell Populations in Antibody Mediated Rejection

**DOI:** 10.1007/s40472-026-00537-4

**Published:** 2026-11-06

**Authors:** Eugenia Karulina, Michael Nicosia

**Affiliations:** 1 Department of Microbiology and Immunology, Columbia University, New York City, NY USA; 2 Florida Research and Innovation Center, Cleveland Clinic Research, Port St. Lucie, FL USA

## Abstract

**Purpose of Review:**

Antibody-mediated rejection (ABMR) is a leading cause of late graft loss, and current therapies act almost exclusively on the effector arm of the alloimmune response. This review examines three classes of regulatory cell from innate and adaptive lineages and the stages of the ABMR response at which each acts, in order to assess their potential as therapeutic targets.

**Recent Findings:**

CD4⁺ Tregs constrain alloimmune priming through CTLA4 dependent costimulatory blockade, cytokine-mediated suppression, and competition for IL-2. CXCR5⁺CD8⁺ antibody-suppressor cells directly eliminate donor-reactive IgG⁺ B cells in murine models. T follicular regulatory (Tfr) cells restrain germinal center reactions and alloantibody generation, and their loss produces enhanced, more pathogenic donor-specific antibody (DSA). Notably, Tfr regulation is initiated early in T-B interactions, Tfr expansion physiologically lags that of Tfh cells, and Tfr numbers are reduced in chronic ABMR. Regulatory B cells and myeloid-derived suppressor cells remain comparatively unexplored in ABMR, and the latter carry a risk of profibrotic differentiation under chronic inflammatory conditions.

**Summary:**

Regulatory cell populations act predominantly during the early phases of the alloimmune response. Their therapeutic utility in ABMR is therefore likely to be constrained by timing, and to lie in the prevention of de novo DSA rather than the reversal of established rejection.

## Introduction

In the US alone over 48,000 life extending solid organ transplants were performed in 2024 for patients with end-stage kidney, liver, heart, lung, pancreatic or intestinal disease [1]. While 1- and 3-year survival rates are excellent, long term graft attrition remains a major concern. A major contributor to late graft loss is antibody mediated rejection (ABMR) driven by donor specific antibodies (DSA) that recognize donor major histocompatibility complex (MHC) molecules, as well as incompatible ABO blood group antigens.

ABMR causes significant acute and chronic graft injury and arises from dynamic interaction between innate and adaptive immune responses. Dendritic cells present donor-derived MHC and non-MHC peptides to recipient T cells, a subset of which differentiate into allospecific follicular helper T (Tfh) cells. These Tfh cells provide help to germinal center (GC) B cells, driving GC reactions resulting in the generation of DSA producing plasma cells. Binding of circulating DSA to endothelial MHC initiates two principal mechanisms of ABMR pathogenesis. DSA can induce endothelial cell injury directly via the fixation of complement and the resulting recruitment of innate immune cells that further contribute to local inflammation and donor endothelial cell injury [2, 3]. Additionally, DSA binding in the graft microvasculature can induce the activation of NK cells. NK cell antibody-dependent cellular cytotoxicity (ADCC) is mediated through the expression of CD16a/FCγRIIIa. Engagement of this receptor has been shown to trigger the release of interferon-gamma (IFNγ) and enhance cytotoxicity, leading to localized tissue damage, and diminished graft function [4].

**Fig. 1:**
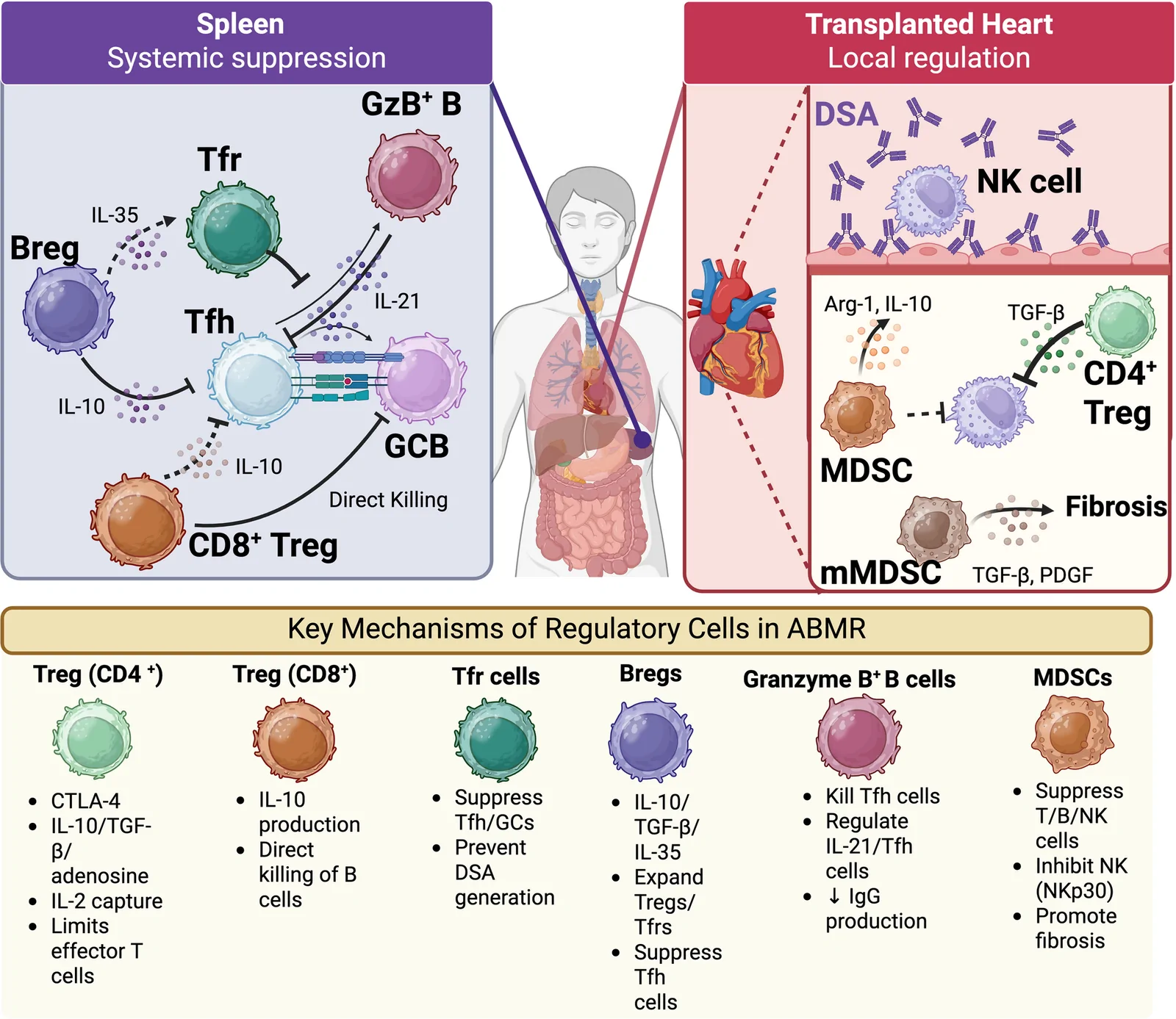
Regulatory immune cell populations in antibody-mediated rejection (ABMR). Regulatory cells act in two compartments. In the spleen (systemic suppression), they restrain the germinal center response generating donor-specific antibody (DSA): Bregs suppress T follicular helper (Tfh) and germinal center B cells (GCB) and expand T follicular regulatory (Tfr) cells via IL-10, IL-35, and TGF-β; Tfr cells suppress Tfh/GCs; CD8⁺ Tregs suppress Tfh (IL-10) and kill GCB; granzyme B⁺ B (GzB⁺ B) cells kill Tfh and reduce IgG. In the transplanted heart (local regulation), DSA engages endothelium and activates NK cells; CD4⁺ Tregs suppress effector responses via TGF-β, and MDSCs suppress T/B/NK cells (Arg-1, IL-10). MDSCs are dual-function: monocytic MDSCs (mMDSC) can also promote fibrosis (TGF-β, PDGF). The lower panel summarizes each population’s principal mechanisms. Solid lines, proven effects; dashed lines, proposed effects. Created in BioRender. Nicosia, M. (2026) https://BioRender.com/b0g008x

One of the major challenges in preventing ABMR has been the paucity of effective treatments. Complement inhibitors [5, 6], plasmapheresis, and proteasome inhibitors [7, 8] are all limited in their efficacy and are often accompanied by undesirable side effects [9]. Even daratumumab – an anti-CD38 depleting antibody that can deplete recipient NK and plasma cells critical in ABMR progression and pathology - lacks allo-specificity and leaves patients susceptible to infection due to the broad clearance of antibody producing plasma cells [10]. Historically treatments in transplantation have targeted a single arm of the immune response – the effector response. By contrast the therapeutic potential of regulatory cells remains largely unharnessed by current regimens. In this review we examine three classes of regulatory cell; regulatory T cells, regulatory B cells, and myeloid-derived suppressor cells and the stages of the ABMR response at which each acts, from alloimmune priming and germinal center reactions through to antibody-mediated effector injury (Fig. 1). In doing so we highlight both the therapeutic potential of these populations and the temporal constraints that will govern their clinical application.

## Regulatory T Cell Populations

Regulatory T cells (Tregs) are suppressive lymphocytes that limit immune activation and maintain tolerance. Treg subsets differ in developmental origin, phenotype and localization. The most well-defined Treg population is composed of CD4⁺CD25^hi^CD127^lo^FOXP3⁺ cells. FOXP3 is a key Treg transcription factor that maintains the core regulatory program. CD25 is a subunit of the high-affinity receptor for interleukin-2 (IL-2), a cytokine required for Treg survival. Tregs can be thymus-derived (tTregs), peripherally derived (pTregs), or generated in vitro (iTregs). Other Treg populations include CD8⁺ Tregs and T follicular regulatory (Tfr) cells. These subsets may control different stages of the alloimmune response that leads to DSA formation and ABMR [11, 12].

### CD4 Tregs

Most stable, conventional CD4⁺ Tregs develop in the thymus and are called tTregs, historically termed natural Tregs (nTregs). pTregs arise from naïve conventional CD4⁺ T cells after peripheral antigen recognition when local antigen-presenting and cytokine signals favor FOXP3 induction over differentiation into effector T cells. Such conditions include limited costimulation and signaling through TGF-β and IL-2. TGF-β promotes FOXP3 expression, and IL-2 supports Treg survival and stability. These populations can share surface markers but differ in their stability, or in other words resistance to pro-inflammatory reprogramming [11, 12].

CD4⁺ Tregs have the potential to regulate ABMR during alloimmune priming, DSA development, and antibody-associated graft inflammation. The earliest point of intervention is indirect allorecognition. In this pathway, recipient antigen-presenting cells (APCs) process donor proteins and present donor peptides to recipient CD4⁺ T cells. These helper T cells can later support donor-reactive B cells. Tregs express cytotoxic T lymphocyte associated protein 4 (CTLA4), an inhibitory receptor that reduces the availability of CD80 and CD86 on APCs. Because CD80 and CD86 provide signals needed for conventional T cell activation, their removal could limit the expansion of donor-reactive helper T cells [13, 14].

Tregs can also reduce local inflammation by producing immunosuppressive cytokines. Treg-derived IL-10 suppresses inflammatory cytokine production and antigen presentation by myeloid cells. TGF-β limits the activation of several lymphocyte populations. Some Tregs express CD39 and CD73, enzymes that convert pro-inflammatory extracellular ATP and ADP released from injured tissue into immunosuppressive adenosine. These mechanisms could reduce endothelial inflammation after DSA binds to the graft, even though they do not neutralize the antibody itself. Tregs are not known to directly block complement proteins; however, reduced DSA production would be expected to decrease antibody deposition and therefore reduce activation of the complement pathway [12].

Tregs produce little IL-2 because FOXP3 suppresses transcription of the IL-2 gene [15]. They therefore depend mainly on IL-2 released by nearby activated effector T cells [16]. Tregs constitutively express high levels of CD25, which combines with the signaling subunits CD122 and CD132 to form a high-affinity IL-2 receptor, allowing Tregs to capture IL-2 efficiently [17]. The resulting IL-2/STAT5 signaling supports Treg survival and maintains FOXP3 expression and suppressive function [18, 19]. Efficient IL-2 capture also lowers the concentration of free IL-2 in the surrounding environment. Because recently activated effector T cells require IL-2 to support their continued proliferation and survival, competition for IL-2 can restrict their expansion. In one study, Treg-mediated cytokine deprivation induced apoptosis of effector CD4⁺ T cells both in vitro and in a mouse model of intestinal inflammation [20]. In ABMR, this competition could limit the expansion and persistence of donor-reactive effector T cells, reducing both graft inflammation and the T-cell help needed to sustain pathogenic alloantibody responses.

Human studies indicate that both Treg abundance and localization are altered during ABMR. Louis et al. found persistent reductions in circulating Tregs in kidney-transplant recipients with ABMR [21]. In contrast, Bunnag et al. detected increased FOXP3 expression in rejecting kidney grafts, including grafts with ABMR, but higher intragraft FOXP3 expression was not associated with better graft outcomes [22]. These data must be interpreted with the caveat that activated effector T cells can transiently express FOXP3. Nevertheless, the findings suggest that reduced Treg numbers may reflect impaired generation, effectiveness or localization, while intragraft FOXP3 accumulation may represent a compensatory response that is too late or insufficient to control established rejection.

Liao et al. provide more direct evidence in a mouse kidney transplant model, administering TGF-β-induced iTregs to presensitized recipients. Treatment reduced circulating DSA, Tfh cells, B cells, and plasma cells. It also reduced graft IgG and C3d deposition, likely reflecting reduced antibody deposition rather than direct complement inhibition, which was not tested. Microvascular inflammation, natural killer (NK) cell infiltration, and inflammatory macrophages were also reduced. These results indicate that iTregs can affect both DSA production and the inflammatory phase of ABMR [23].

Clinical studies show that Treg therapy can be administered safely in transplant recipients. Harden et al. infused autologous polyclonal Tregs into 12 living-donor kidney recipients. The treatment produced a sustained increase in circulating Tregs and did not increase adverse events. All treated recipients remained free of acute rejection over four years, compared with 78.9% of standard-care recipients. DSA testing was negative in the treated patients who were tested at 60 weeks. However, the cohort was small, non-randomized, and mainly nonsensitized. This study supports the safety and persistence of Treg therapy but does not address its ability to treat established ABMR [24].

### CD8 Tregs

CD8⁺ Tregs are less well-defined than CD4⁺FOXP3⁺ Tregs. They do not arise from any one recognized developmental pathway and lack a single lineage marker. Reported populations include CD8⁺FOXP3⁺, CD8⁺CD28⁻, CD8⁺CD122⁺, and CD8⁺CXCR5⁺ cells. Some appear to develop during thymic selection, while others are induced from conventional CD8⁺ cells by antigen exposure, chronic stimulation, or homeostatic cytokines. CD122 is the shared receptor subunit for IL-2 and IL-15, and is associated with a memory-like CD8⁺ regulatory population. Since these markers are also found on non-regulatory CD8⁺ cells, suppressive activity must be demonstrated [25]. CD8⁺CD122⁺ Tregs may suppress alloimmunity through IL-10 production. In a mouse allograft model, these cells produced more IL-10, inhibited conventional T-cell proliferation more strongly, and prolonged graft survival more effectively than CD4⁺CD25⁺ Tregs. Loss of IL-10 reduced their suppressive advantage. However, the study did not directly examine DSA formation or ABMR [26].

CXCR5⁺CD8⁺ antibody-suppressor cells may have a more direct connection to humoral rejection. In a mouse kidney transplant model, donor-primed CXCR5⁺CD8⁺ cells showed cytotoxic activity against donor-activated IgG⁺ B cells. Adoptive transfer reduced alloantibody, improved ABMR-like pathology, and prolonged graft survival. CXCR5-negative cells and cells primed against unrelated donor antigens were ineffective, indicating that both follicular localization and donor specificity were required. The exact molecular pathway responsible for B-cell killing was not fully defined [27]. Han et al. later showed in another mouse kidney transplant model that CXCR5⁺CD8⁺ cells reduced donor-reactive germinal center B cells, alloantibody, and ABMR-like injury more effectively than conventional CD4⁺ Tregs. T cell receptor transgenic experiments confirmed antigen-specific activity. These findings support direct targeting of the donor-reactive B cell response. However, it remains unclear whether humans have a comparable CXCR5⁺CD8⁺ population that specifically targets donor-reactive B cells and suppresses alloantibody production [28].

### T Follicular Regulatory Cells

Although some DSA are generated extrafollicularly, the majority arise in the secondary lymphoid organs as a result of interactions between Tfh cells and GC B cells. These interactions, and the resulting germinal center reactions induce clonal proliferation, class switch recombination and somatic hypermutation, thereby augmenting the pathogenic potential of the antibodies [29, 30]. Follicular regulatory T (Tfr) cells are a specialized subset that arises from tTregs and shares substantial phenotypic overlap with Tfh cells. Like Tfh cells, they express the transcription factor Bcl6 and the chemokine receptors CXCR5 and CXCR4 that facilitate follicular entry, but they retain FOXP3 expression and thus regulatory function [31, 32]. Tfr cells additionally express regulatory molecules such as CTLA-4, PD-1, and LAG3 [33–35]. Mice deficient in Tfr cells develop self-reactive plasma cells following influenza infection, and spontaneously develop autoantibodies with age, suggesting a critical role in the maintenance of tolerance [36–38]. In addition to suppressing antibody production, Tfr cells promote clonal diversity and affinity maturation, suggesting a complex role in the regulation of antibody responses [39].

The ability of Tfr cells to regulate germinal center reactions places them as key regulators of the ABMR response and of DSA generation. Tfh cells provide B cells with CD40-dependent costimulation and interleukin-21 (IL-21). IL-21 promotes B cell proliferation, class switching, and differentiation into antibody-secreting plasma cells. Tfr cells limit this process by suppressing both Tfh cells and B cells. They reduce Tfh expansion and cytokine production, including IL-21 [34]. B cells exposed to Tfr cells in vitro showed reduced glucose metabolism, class-switch recombination, and antibody production. These changes persisted after the Tfr cells were removed. These effects were associated with changes in chromatin accessibility, suggesting that Tfr cells partly maintain B cell suppression by altering the expression of genes involved in metabolism and antibody production [40]. In ABMR, these effects could reduce the generation of DSA.

Their role in regulating alloantibody responses is only beginning to be defined. Zhang et al. recently demonstrated that Tfr cell deficiency permitted a robust DSA response culminating in acute ABMR, indicating that Tfr cells actively restrain alloantibody production [41]. They further identified a link between follicular dysregulation and cellular inflammation. Germinal center B cells from Tfr-deficient mice expressed more IL-15, a cytokine that can support T cells, NK cells, and B cell responses. When IL-15 was neutralized with a blocking antibody, germinal center B cell and DSA numbers were reduced, and graft survival was prolonged. Tfr cells therefore appear to restrain both antibody production and inflammatory B cell programming by limiting IL-15-dependent amplification of the alloimmune response [41]. Early Tfr depletion posttransplant increased germinal center B cells, memory-like B cells, donor-reactive B cell clones, and circulating DSA. It also increased glomerulitis, peritubular capillaritis, and graft C4d deposition. To test the pathogenicity of the antibodies produced, the authors transferred serum into kidney-transplanted mice that lacked mature B cells. Serum from Tfr-deficient recipients caused greater graft IgG and C4d deposition, indicating that the antibodies generated in the absence of Tfr cells are more pathogenic [41].

Human evidence links an altered Tfh/Tfr balance to chronic ABMR. Chen et al. found fewer circulating and intragraft Tfr cells in kidney recipients with chronic ABMR. These patients also had more IL-21-producing Tfh2- and Tfh17-like cells. Tfr levels were inversely correlated with DSA levels, and IL-21-producing Tfh subsets correlated positively with DSA. The remaining Tfr cells retained suppressive activity in vitro: increasing the proportion of Tfr cells reduced B-cell proliferation, plasma-cell differentiation, and IgG production. This suggests that insufficient Tfr numbers or localization, rather than loss of function, may be the more relevant defect [42].

Mohammed et al. used an inducible Tfr deletion model in mice to test whether Tfr cells directly restrain alloantibody formation after kidney transplant. Removing Tfr cells increased de novo IgG DSA, showing that Tfr cells normally suppress the development of donor-reactive B cell responses. However, Tfr deletion caused only a modest additional increase in graft rejection, possibly because the fully MHC-mismatched grafts were already rejected rapidly. The study therefore provides causal evidence that Tfr cells limit DSA production, but only limited evidence that Tfr loss worsens graft survival [43].

The reason for low Tfr numbers in ABMR remains unknown. Immunosuppressive treatment may contribute, because lymphocyte-depleting therapies can cause long-lasting changes in circulating Tfr populations [44]. Tfr instability may provide another explanation. Mouse fate mapping studies have shown that Tfr cells pass through several differentiation states and can lose FOXP3 and become ex-Tfr cells, potentially acquiring an effector helper T-cell-like transcriptional profile. Human lymph node Tfr cells were found to contain a similar instability-associated transcriptional profile [45]. Tfr development timing may also matter. In a mouse kidney transplant model, Tfh and germinal center B cells expanded before Tfr cells, creating an early window in which B cell help exceeded regulation. This may contribute to reduced Tfr numbers in ABMR, and introduces a key temporal dimension to Tfr based intervention [41].

Clement et al. used the NP-OVA immunization model to track antigen-specific B cell and antibody responses in mice. Removing Tfr cells early after immunization increased antigen-specific B cell expansion, plasmablast formation, memory B cell development, and antibody production before mature germinal centers formed. Removing Tfr cells later had substantially less effect. This suggests that Tfr-mediated regulation begins during early T/B-cell interactions rather than after a germinal center response is established [46]. By extension, Tfr cells may limit the number of donor-reactive B cells that receive enough antigen and Tfh-derived help to enter germinal centers, expand, undergo class switching and somatic hypermutation, and differentiate into memory B cells or plasma cells. Sage and Sharpe proposed that plasma cells may become less accessible to Tfr regulation after migrating from lymphoid organs to bone marrow survival niches, although this has not been directly demonstrated [34]. In transplantation, these findings suggest that Tfr cells may be better positioned to prevent early donor-reactive B cell responses than to control established DSA production.

Any Tfr-directed therapy must therefore account for timing. Tfr treatment may be better suited to preventing de novo DSA than reversing chronic ABMR maintained by long-lived plasma cells. IL-21 is produced by activated Tfh cells and reinforces follicular antibody responses by supporting Tfh differentiation and B cell activation. It can also inhibit FOXP3 expression, limiting Treg differentiation. Nian et al. tested whether blocking the IL-21 receptor could shift the follicular response away from Tfh-mediated help and toward Tfr-mediated regulation. In vitro, an anti-IL-21R antibody inhibited the differentiation of naïve CD4⁺ T cells into CXCR5⁺ Tfh cells and reversed the reduction in FOXP3⁺ Treg differentiation caused by IL-21. They then transplanted fully MHC-mismatched BALB/c skin grafts onto C57BL/6 mice and administered anti-IL-21R beginning on the day of transplantation. After 14 days, treated mice had fewer activated CXCR5⁺ICOS⁺PD-1⁺ Tfh cells, more CXCR5⁺FOXP3⁺ Tfr cells, and a lower Tfh: Tfr ratio in the spleen. They also had fewer germinal center B cells and reduced germinal center formation. Donor-specific IgG was monitored for five weeks and was significantly lower at week three, although donor-specific IgM was unchanged. These findings show that early IL-21R blockade can alter the developing follicular response and reduce de novo class-switched DSA [47].

Conventional CD4⁺ Tregs and Tfr cells must not be viewed as redundant: they appear to regulate ABMR through overlapping temporal windows but by distinct mechanisms. CD4⁺ Tregs can influence the development of the humoral response by limiting antigen-driven T-cell activation and Tfh-cell formation, including through regulation of costimulatory signals. In mice, depletion of Tregs during the primary immune response to immunization increased antigen-specific Tfh cells, germinal-center responses, plasma cells, and memory B cells, and enhanced the subsequent secondary antibody response [48]. In mouse allograft models, donor-specific CAR-Tregs reduced DSA and DSA-secreting B cells in naïve recipients but failed to suppress established humoral alloimmunity in sensitized recipients [49]. Tfr cells, in contrast, act within the follicular compartment, where they constrain Tfh–B-cell interactions and suppress B-cell proliferation, class-switch recombination, metabolism, and antibody production [40, 50]. Tfr regulation is particularly important during the early B-cell response, as Tfr depletion has a greater effect when performed before GC maturation [46]. Thus, both CD4⁺ Tregs and Tfr cells appear to limit the development of de novo DSA, but CD4⁺ Tregs act more broadly on the alloimmune response, whereas Tfr cells provide specialized control of donor-reactive B cells within the follicular compartment. Neither population is likely to control established humoral alloimmunity once long-lived antibody-producing cells have developed.

## Regulatory B Cell Populations

Regulatory B cells (Bregs) were initially characterized as IL-10-producing B cells [51], but recent studies have revealed a biology considerably more complex and nuanced than first conceived. Bregs are a collection of B cell subsets that lack a canonical marker like FOXP3 to define them [52]; instead, several characteristics can identify a cell as a Breg. TIM-1 is the closest to a universal Breg marker with it being expressed by approximately 75% of IL-10^+^ B cells [53]. Other Breg effector molecules include TGF-β, IL-35 and even granzyme B [54–56]. While Bregs are important in regulating several other aspects of the posttransplant immune response, there is limited direct data on their involvement in ABMR. However, IL-10 production directly suppresses CD4 T cell and Tfh cell function [57], and stimulates Treg expansion, which could in turn contribute to regulation of ABMR [58]. The Breg cytokine, IL-35 has been shown to expand Tfr numbers skewing the Tfh: Tfr balance toward a more tolerogenic environment [59]. However, these studies did not identify the cellular source of IL-35, which could equally be produced by Tregs or tolerogenic DCs; as such, the role of IL-35 producing Bregs in the regulation of ABMR has yet to be explored.

### Granzyme B Producing B Cells

Clinical studies have identified increased frequencies of highly differentiated granzyme B^+^ B cells in operationally tolerant kidney transplant recipients [60]. Regulation by these cells is dependent on granzyme B production, and on contact with target cells. In an apparent feedback loop, the number of granzyme B^+^ plasma cells is dependent on IL-21 production, and these cells control the numbers of IL-21 producing Tfh cells and the amount of IL-21 in the system [61–63]. Clinically, methods have been developed to expand these cells ex vivo, as these cells have been shown to demonstrate transferable tolerance in rat transplant models [64].

### IL-10 + Plasma Cells

A recently published study investigating the role of the coinhibitory receptor LAG3 in ABMR, found LAG3 expression on plasma cells, and the loss of LAG3 systemically, restricted to cells of the B cell lineage, or via a blocking antibody resulted in enhanced alloantibody responses [35]. The authors found that IL-10 expression in plasma cells was dependent on LAG3, though it remains unclear if this is due to a developmental defect in IL-10^+^ plasma cells in the absence of LAG3 signaling, or whether LAG3 signaling is the trigger for IL-10 production by these cells. However, their data show that LAG3 engagement diminished IgG production suggesting that engagement of LAG3 on IL-10^+^ plasma cells mediates regulation by these cells [35].

## Myeloid Derived Suppressor Cells

Monocytes and neutrophils with immunosuppressive functions were first reported in the 1990s, and as the field developed, were later renamed myeloid derived suppressor cells (MDSCs) [65]. MDSCs are divided into two major groups, granulocytic/polymorphonuclear MDSCs (gMDSCs/PMN-MDSCs) and monocytic MDSCs (mMDSCs), based on their lineage. These cells arise from common myelopoiesis, at an intersection of pathological activating signals associated with persistent inflammation that includes elevated levels of GM-CSF, IL-6, and IL-1β [66]. MDSCs are potent suppressors of T cells, B cells and NK cells [66], with gMDSCs utilizing reactive oxygen species, arginase 1 and prostaglandin E2, and mMDSCs employing more conventional immunosuppressive cytokines such as IL-10 and TGF-β [67]. MDSC studies in the context of ABMR are sparse, but data from cancer and fibrotic disease suggest a plausible role for MDSCs in ABMR progression.

In tumors, MDSCs are potent suppressors of Fc-receptor mediated functions in NK cells, specifically preventing ADCC and IFNg production [68]. The mechanism of this suppression remains contested: one study proposed it is via MDSC production of iNOS and arginase through a mechanism that requires cell to cell contact [68]. A study contested this mechanism, but did agree that cell to cell contact was a requirement for MDSC suppression of NK cell function [69]. Instead, Hoechst and colleagues showed that blocking NKp30, a major activating receptor of NK cells [70], reduced NK cell mediated cytolysis and IFNγ production [69]. The effect of NKp30 blockade was dependent on the presence of MDSCs, suggesting that NKp30 engagement is a key pathway in MDSC-mediated inhibition of NK cell function. Given the importance of DSA induced NK cell ADCC within the graft microvasculature [2–4], these findings from the cancer field underscore the need for further exploration into the potential of MDSCs to suppress NK cells during the effector phase of ABMR.

However, leveraging MDSCs in ABMR may be a double-edged sword. Studies have shown that the mMDSC subset is highly plastic and can differentiate into profibrotic, M2-like macrophages following chronic exposure to cytokines such as IL-6 and IL-10 [71], conditions that mirror those within a chronically rejecting allograft. This transition leads to the secretion of TGF-β and platelet-derived growth factor (PDGF), which drive fibroblast to myofibroblast and fibroblast proliferation respectively [72, 73], a process that would be deleterious in the long term in an allograft. One of the hallmarks of MDSCs is high expression of arginase-1 (Arg-1), which serves as a mechanism to starve T cells by depleting L-arginine from the local environment [74, 75]. Studies of lung fibrosis have shown that Arg-1 drives fibrosis via the conversion of arginase ◊ ornithine ◊ proline, a key substrate for collagen synthesis [76]. These pathways, although unexplored in the context of chronic ABMR (cABMR), could prove to be significant hurdles in leveraging the suppressive potential of MDSCs.

## Interaction of Regulatory Cells with Immunosuppression

An optimal therapeutic approach is one that restrains effector cells and thus limit graft injury, while promoting the survival and function of regulatory cell subsets. Calcineurin inhibitors (CNIs) such as tacrolimus and cyclosporin are potent suppressors of effector T cells primarily by blocking NFAT resulting in the reduction of IL-2 production [77]. The most potent effect of CNIs is on Tregs as IL-2 is a critical factor for Treg survival and thus CNIs compromise the immunoregulatory capacity of Tregs and promote the apoptosis of both resting and activated Treg subsets [78]. It is likely these mechanisms impact Tfr cells in a similar manner given the shared importance of IL-2 signaling, thought there is currently no experimental data to support this. CD8 Tregs differ on their IL-2 dependance by subset and the effects of CNIs on their function remains to be explored [79]. CNI-induced changes in T cell function and thus cytokine milieu can support the development of regulatory B cells, however the direct effects of CNIs have not been studied [80]. In MDSCs CNIs have been shown in mouse skin transplant models to increase the numbers of MDSCs in the graft and induces their expression of IDO, limiting T cell access to tryptophan further limiting their function [81].

Belatacept and abatacept are CTLA-4-Ig fusion proteins that sequester CD80/CD86 and withdraw the CD28 signals on which suppressive lymphocytes depend. They are predominantly detrimental to regulatory T-cell populations in transplantation. CTLA4-Ig contracts the CD4⁺ Treg pool in murine allograft models [82], and Tregs recovered from long-term belatacept-treated kidney recipients carry both intrinsic defects (impaired CD25/IL-2 responsiveness) and extrinsic defects despite phenotypic and functional recovery ex vivo [83]. Contraction of the Treg pool is widely implicated in the elevated acute rejection rate observed with belatacept, with adjunctive IL-2 the principal strategy proposed to restore it [84]. The germinal-center axis is similarly suppressed, belatacept reducing Tfh frequency, activation, and alloantibody help [85], although follicular regulatory T cells themselves remain poorly characterized in this setting. No transplant data support expansion of CD8⁺ regulatory subsets. The notable exception is the regulatory B-cell compartment, which is favored: belatacept promotes accumulation of CD24^hi^CD38^hi^ transitional B cells, lowers BAFF, and induces STAT3-dependent IL-10⁺ Bregs [86, 87], correlating with reduced donor-specific antibody formation, though transitional-B expansion is not uniformly observed. Data on myeloid-derived suppressor cells under CTLA-4-Ig in transplantation are effectively absent.

mTOR inhibition is a potent inhibitor of effector T cells due to their mTOR dependent metabolic and proliferative requirements, however CD4 Tregs tolerate mTOR inhibition well resulting in increased Treg: effector ratio and increased Treg suppressive function [88]. Likewise, rapamycin has been shown to favor the enrichment of human CD103^+^CD8^+^ Tregs in vitro and the enhancement of their suppressive function [89], however there are no supporting reports that show this in vivo. There is little evidence of the effect of mTOR inhibition on Tfr cells however the contraction of effector T cells and the increased ratio of Tregs: effectors could favor the increase in Tfr numbers and function. In Bregs the data are mixed where one clinical kidney transplant study showed mTOR treated recipients had reduced Breg populations [90], while reports from liver transplant patients show that CD19^+^CD24^+^CD38^+^ Breg numbers and function expanded following switching treatment from tacrolimus to sirolimus [91]. More studies are needed to dissect the mechanisms and interrogate which Breg subsets are responsive. In MDSCs mTOR inhibition is heavily context dependent. Some studies do show that MDSCs are induced under Rapamycin treatment, and this contributes to allograft survival in mouse heart transplant models, while other studies show a contraction of the MDSC pool following mTOR inhibition [92, 93]. Variables such as which MDSC subset, the tissue examined, dose and duration of Rapamycin treatment and degree of inflammation all impacting the overall picture.

Corticosteroids are broad suppressors of inflammation by preventing transcription, cytokine production and antigen presentation, as a result can suppress the proliferation, phenotype and function of CD4 + Tregs [94]. The evidence in CD8 + Tregs, Tfrs and Bregs is limited, and should be considered broadly suppressive in the context of Tfh-GCB interactions but direct association with CD8^+^ Tregs, Tfrs and Bregs has not been proven [95, 96]). Corticosteroids have been shown to benefit the function of MDSCs. Following dexamethasone treatment, the recruitment/induction of iNOS-expressing suppressive MDSCs was increased [92]. However, dexamethasone treatment is not common in transplant patients compared to methylprednisone (MP) and prednisone, and while MP facilitates expansion of MDSCs ex vivo [97], preclinical in vivo or clinical data is scarce.

Other immunosuppressives that are commonly used in transplant have even less evidence linking them to a direct effect on regulatory cell function save Basiliximab a CD25 blocker, the receptor CD4^+^ Tregs rely on for survival [94, 98]. Largely, more research is required to investigate the effects of current immunomodulatory drugs on regulatory immune cells outside of the classical CD4 expressing Treg. Understanding these effects may better guide clinical decision making on how and when to use them.

## Conclusion and Future Directions

Current therapies for ABMR (complement inhibitors, plasmapheresis, proteasome inhibitors and anti-CD38 depleting antibodies) act on the effector arm of the alloimmune response, lack allospecificity, and are of limited efficacy. Regulatory cell populations offer an alternative point of intervention, acting upstream to constrain the generation of donor-reactive B cell responses rather than removing their products.

The studies reviewed here indicate that the principal constraint on this approach is temporal. CD4⁺ Tregs, CXCR5⁺CD8⁺ antibody-suppressor cells and Tfr cells each suppress donor-reactive B cell responses in preclinical models, but the interval during which they are effective appears to be early. Tfr-mediated regulation is initiated during early T-B interactions rather than after germinal center establishment [46], Tfr expansion lags that of Tfh and germinal center B cells, generating a window of unopposed help [41], and plasma cells that have migrated to bone marrow niches may be inaccessible to follicular regulation [34]. Consistent with this, adoptive Treg therapy is safe and persistent in nonsensitized recipients [24] but has not been shown to reverse established ABMR. MDSCs are subject to an analogous constraint: their capacity to suppress NK-mediated effector injury is offset by differentiation into profibrotic macrophages under conditions of chronic inflammation.

Three questions require resolution. First, whether human equivalents of the CXCR5⁺CD8⁺ and IL-10⁺ plasma cell populations exist, and whether they retain suppressive function. Second, the basis of Tfr deficiency in ABMR: depletion by induction therapy, FOXP3 instability and delayed development are not mutually exclusive, but they imply different interventions. Third, whether recipients at risk of de novo DSA can be identified prior to seroconversion, since the utility of any regulatory cell therapy will depend on its administration within this window.

## Data Availability

No datasets were generated or analysed during the current study.
